# Retrospective Approach to the Endemic *Dianthus fruticosus* L. ssp. *fruticosus* on Serifos Island (Cyclades, Greece)

**DOI:** 10.3390/plants13213002

**Published:** 2024-10-27

**Authors:** Sophia Rhizopoulou, John Pouris

**Affiliations:** Section of Botany, Department of Biology, National and Kapodistrian University of Athens, 15784 Athens, Greece; jopouris@biol.uoa.gr

**Keywords:** archives, carnation, native, island, seasonality, vegetation history

## Abstract

The carnation *Dianthus fruticosus* L. ssp. *fruticosus* (Caryophyllaceae) is a range-restricted perennial, endemic plant that grows on cliffs, rocks, ravines, terraces, and boulders on Serifos Island in the Cyclades in the Aegean archipelago (Greece), possessing an impressive, aesthetic blossoming during the dry season. This indigenous carnation of Serifos has attracted the interest of naturalists and scientists. Specimens of this subspecific taxon from the island of Serifos (Greece) were collected during botanical explorations in preindustrial times by the French naturalist Joseph Pitton de Tournefort (1702) and in the late eighteenth century by the English Professor of Botany at the University of Oxford John Sibthorp (1787). Those specimens, documented in relevant publications and labelled with different names (i.e., *Caryophyllus Graecus arboreus* and *Caryophyllus arboreus Seriphius* in the early eighteenth century and *Dianthus fruticosus* in the early nineteenth century), are related to aspects of vegetation history, linking the past to the present and (most probably) to the future. Today, a thorough understanding of the in situ development and functionality of this endemic carnation is still required, as is a framework of its sustainability and management in small-scale insular habitats. The timeless perception of the emblematic wild carnation *Dianthus fruticosus* ssp. *fruticosus*, which is still growing on inland and coastal sites of Serifos and neighboring islands in the Cyclades (Greece), is also a reminder that a native plant is often a repository to which local communities may look when crafting their identity.

## 1. Introduction

The genus *Dianthus* L., which belongs to the Caryophyllaceae family, comprises approximately 300 species, among them many range-restricted and/or narrow-endemic species and subspecies in the Mediterranean area [1,2,3]. The southern Mediterranean taxon *Dianthus caryophyllus* is thought to be involved in the ancestry of many cultivated ornamental varieties of carnation [4,5,6,7,8]. Furthermore, considerable (purely aesthetic) interest has been expressed by horticulturists and botanists in new taxa and subspecies indigenous to specific geographic areas of the Mediterranean region [9,10,11]. It was published that in Greece, *Dianthus* is represented by 44 species and 43 subspecies [2]. In another study, 15 species and 24 subspecies of *Dianthus* endemic to Greece were cited [12]. The geographical distribution of 46 *Dianthus* taxa in Greek territories has been published [13].

**Figure 1:**
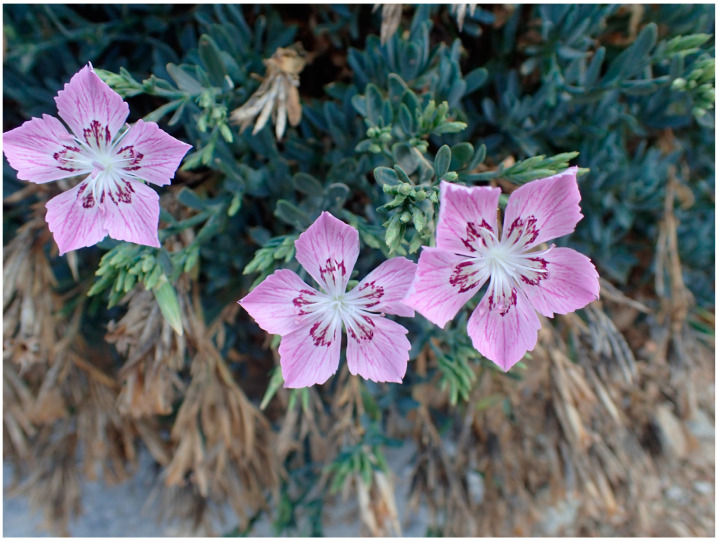
Flowers of *Dianthus fruticosus* ssp. *fruticosus* from Serifos (photo © G. Fassou, reproduced with permission, available at Flora of Greece web) [29].

**Figure 2:**
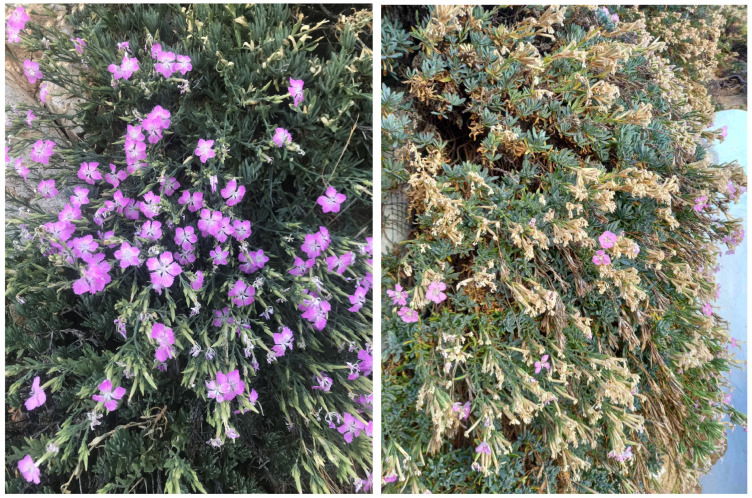
Flowering *Dianthus fruticosus* ssp. *fruticosus* growing on vertical cliffs close to the Chora of Serifos on 27 June (left photo © E. Laskaris, with permission) and on 13 October (right photo © S. Rhizopoulou).

*Dianthus fruticosus* ssp. *fruticosus* (Figure 1) is a range-restricted perennial, endemic subspecies, growing on cliffs, rocks, ravines, terraces, and boulders (Figure 2), distributed on islands of the western and southern Cyclades in Greece [13,14,15,16,17,18]. It is interesting to note that this subspecific taxon is currently characterized as not evaluated according to its IUCN category and as a data-deficient plant according to its IUCN protection status.

*Dianthus fruticosus* ssp. *fruticosus* grows on hard limestone, sunny vertical cliff walls (Figure 2), and crystalline schists on Serifos Island. Its vigorous growth throughout the dry summer and its prolonged flowering (Figure 2) before the raining period point to a water supply during the drought season [14,19,20]. It is well known that water deficiency during summer is considered to be the most stressful environmental factor that affects plant growth and function, which are both controlled by water potential gradients from the soil to the plant and the atmosphere [21,22,23,24]. *Dianthus fruticosus* ssp. *fruticosus*, as well as several other chasmophytes, may utilize water stored in rocks over long periods [14]. The lack of understanding of wild plants’ drought tolerance remains a significant issue linked to the accuracy of modeling future climatic scenarios.

At the international level, there is substantial interest in underutilized plant species as a result of a gradual change in attitude towards biodiversity. In Greece, wild, local, traditional, and neglected plants have recently been included in research plans of the Natural Environment and Climate Change Agency (NECCA), having raised the interest of decision makers.

The touristic status on Serifos Island during summer is linked to substantial water consumption under conditions of water deficiency and human-induced pressure to plant habitats and floristic patterns. In addition, according to the Intergovernmental Panel on Climate Change (IPCC), the frequency and the intensity of extreme weather conditions and climate events, such as heat waves and water deficiency, is expected to increase in some regions, predominantly in the southern and eastern Mediterranean [25,26]. Furthermore, according to the RCP8.5 scenario, the Mediterranean region is expected to receive approximately 20% less precipitation, and this will be a challenge for native, wild plant species.

This work presents an insight into the natural and cultural history of the carnation *Dianthus fruticosus* ssp. *fruticosus* native to Serifos. The nomenclature of this endemic subspecies, an important element of the plant environmental biology of Serifos, has changed over time. However, its current status is linked to long-term survival, early collections, and herbaria’s specimens and archives. This retrospective approach to *Dianthus fruticosus* L. ssp. *fruticosus* contemplates its past situation, botanical history, and current status. *Dianthus fruticosus* ssp. *fruticosus*, native to Serifos Island, is also a reminder that a plant taxon is often a repository at which local communities look when crafting their identities [27]. Elucidating the processes that shape the distribution of an endemic plant is a fundamental challenge and provides tools that allow researchers and policymakers to predict and manage environmental changes. This goal can be achieved through a range of approaches, including research into associations between variables and time and reviewing and synthesizing available information [28].

## 2. Results

### 2.1. The Eighteenth Century

**Figure 3:**
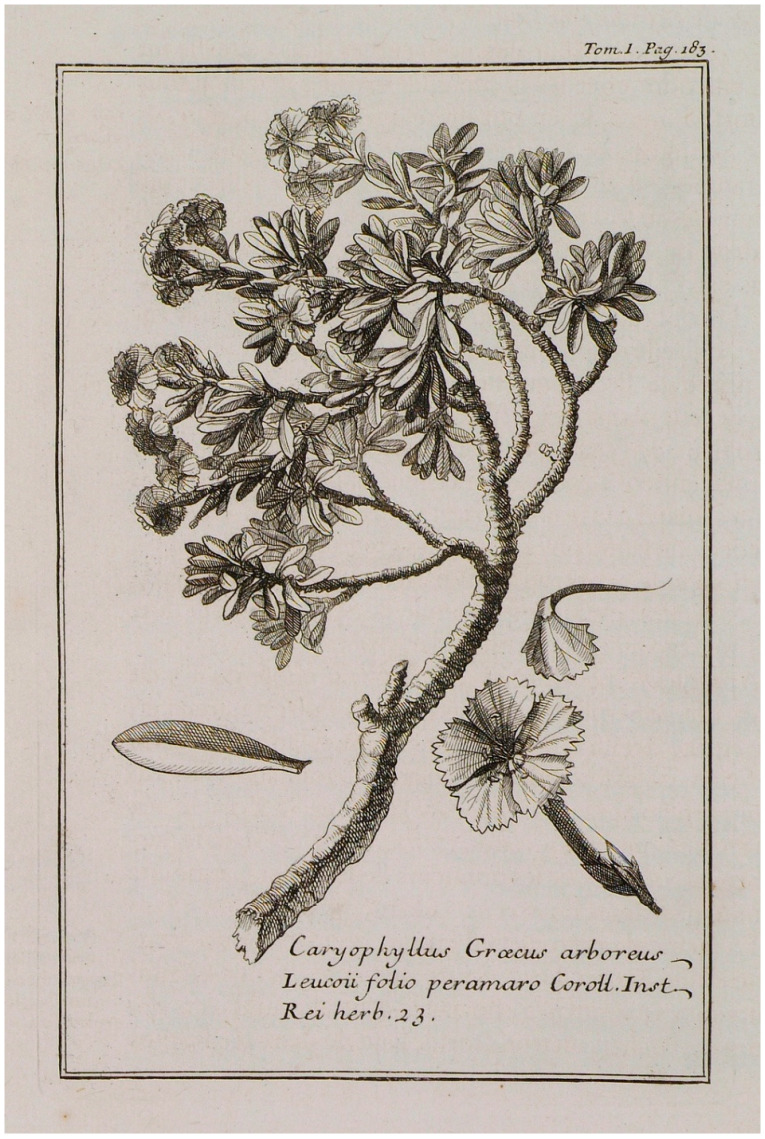
The full-page engraving by Claude Aubriet of the wild carnation collected from Serifos Island by Tournefort and included in the book published in 1717 [31], which was proposed as the typus of *Dianthus fruticosus* [14].

**Figure 4:**
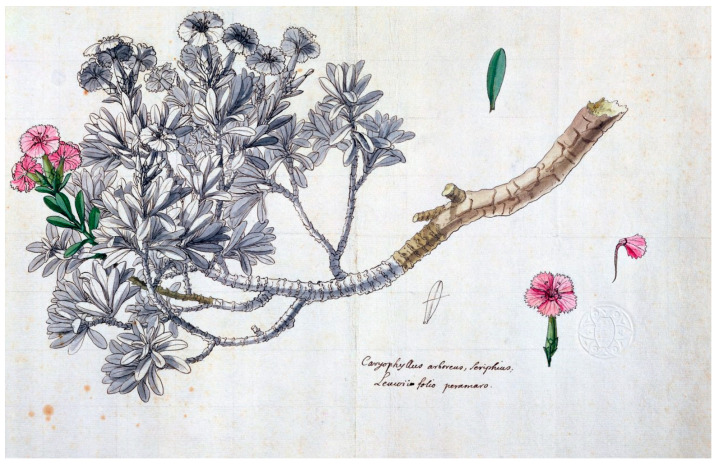
An illustration by Claude Aubriet with a few hand-colored floral, leaf, and stem tissues of *Caryophyllus arboreus Seriphius*, deposited in the Muséum National d’Histoire Naturelle in Paris [32].

The famous French naturalist Joseph Pitton de Tournefort (1656–1708), as part of his botanical exploration of the Levant, visited the islands of the Cyclades in the Aegean archipelago from 1700 to 1702 and collected plants [30]. In the account from his visit to Serifos on 24 August 1700, a detailed description and a full-page engraving [30] (volume I, page 219) by the artist Claude Aubriet (1665–1742) of the endemic carnation were included; the full-page plant engraving was labelled as *Caryophyllus Graecus arboreus* (Figure 3) [30,31,32], which has been proposed as the typus of *Dianthus fruticosus* by Runemark [14]. In addition, an undated, hand-colored illustration portraying the wild carnation by the artist Claude Aubriet and labelled as *Caryophyllus arboreus Seriphius* (Figure 4) was deposited in the Muséum National d’Histoire Naturelle of Paris (France). Comparing Figure 3 with Figure 4, a connection can be seen between them; actually, it seems as they are identical via rotation and inversion, with the exception of the sizes and the positions of the separate drawings of individual flowers, petals, and leaves. The drawing of the wild carnation in Figure 4 probably served as the basis for preparing the engraving in Figure 3; however, relevant information was not found in the literature. It is worth mentioning that illustrations and pictorial representations of plants during the eighteenth century supplemented discoveries and findings with visual evidence; drawings and paintings were functional as identification devices and as a methodological approach to transmitting accurate botanic knowledge, plant diversity, and scientific objectivity [33,34,35,36].

It is noteworthy that the wild carnation of Serifos was labelled in the above-mentioned eighteenth-century illustrations using two different names (i.e., *Caryophyllus Graecus arboreus* and *Caryophyllus arboreus Seriphius*) according to pre-Linnaean nomenclature [37,38,39]; pre-Linnaean polynomial names indicating a given plant usually included several terms used to denote plant features, habitats, and localities, rendering the study of botany difficult. Nevertheless, communication among scientists linked to plant specimens depended on plants’ names; to a certain extent, ordering the names of plants into a classification system, i.e., the eighteenth-century Linnean binomial system, served as a vehicle for the creation of plants’ “identities” [37,40].

John Sibthorp (1758–1796), Professor of Botany at the University of Oxford, being concerned with his botanical reputation and legacy, decided to travel to unexplored areas of the eastern Mediterranean region and Greek territories. In January of 1786, Sibthorp was in Vienna and wrote to his father, “I dream of tracing the steps of Tournefort in the Isles of Archipelago” [41]. Sibthorp’s idea to execute an exploration in the eastern Mediterranean was realized through hard work under field conditions and collection of plant specimens, among them specimens of *Dianthus fruticosus* collected from Serifos (Figure 4) during his botanical exploration from 1786 to 1787 [42].

### 2.2. The Nineteenth Century

**Figure 5:**
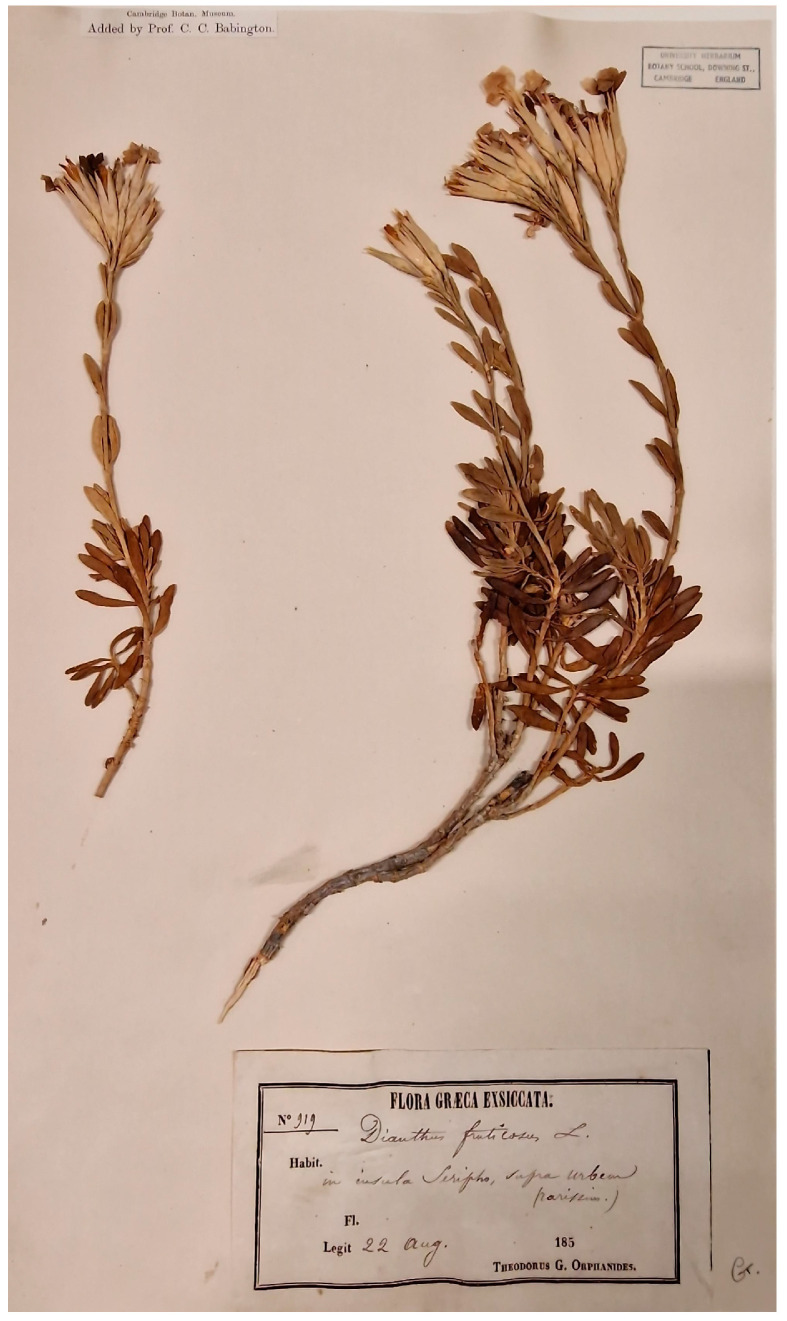
Specimen of *Dianthus fruticosus* L. collected from Serifos Island and housed in the Cambridge University Herbarium (photo © M. del Mar Millan, Herbarium Coordinator at Cambridge University, reproduced with permission).

In 1818, specimens of the wild carnation collected from Serifos by the English banker Stansfield Rawson were deposited in the herbarium of Cambridge University [43]. Stansfield Rawson (1778–1856) was born in Halifax (Yorkshire, UK), and apart from a few details about his family and real estate, no information was found about his journey to Serifos. It is important to note that among the *Dianthus* specimens housed in the Herbarium of Cambridge University (UK), the only *Dianthus fruticosus* that was found (Figure 5) was collected from Serifos (on the label is written “in insula Seripho”) by Theodorus Orphanides (1817–1886), Professor of Botany at the University of Athens in Greece [44,45,46], on 22 August (year unknown) during the nineteenth century (personal communication with Maria del Mar Millan, Herbarium Coordinator at Cambridge University Botanic Garden and Herbarium, on 10 October 2024).

**Figure 6:**
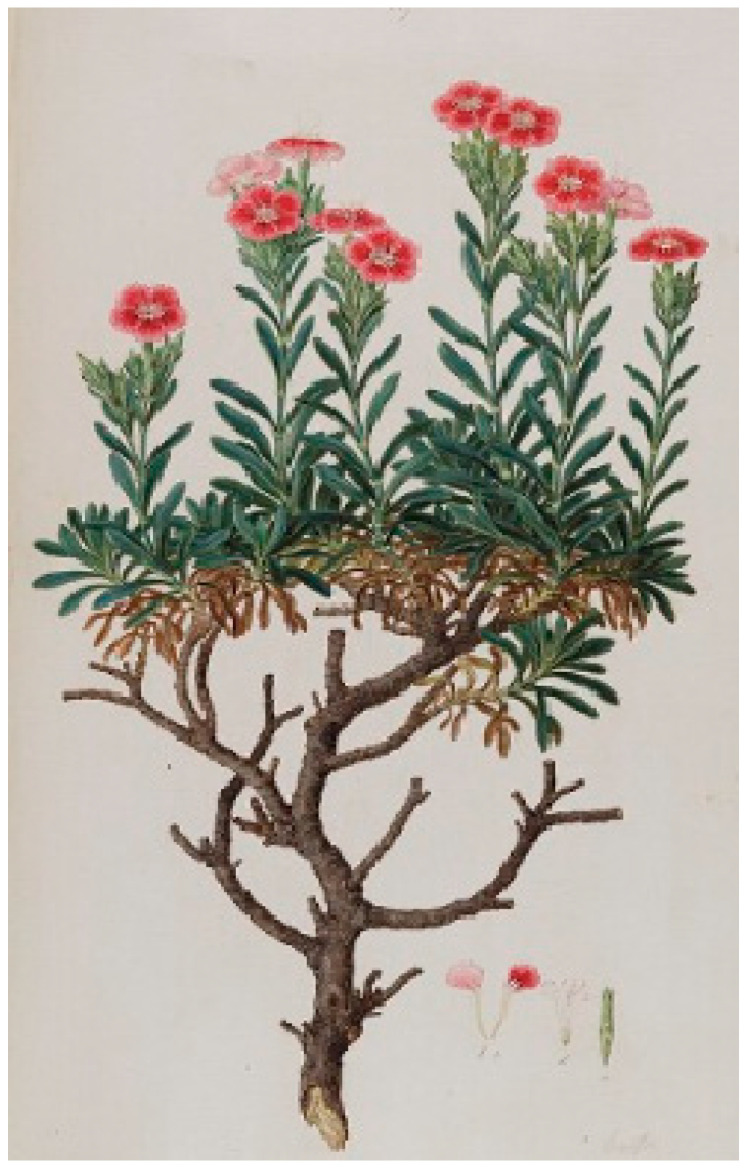
Illustration of *Dianthus fruticosus* collected from Serifos in 1787 and published in *Flora Graeca Sibthorpiana* (1825), based on the original watercolor by Ferdinand Bauer (reproduced with permission of the National Library of Greece).

In the fifth volume of the magnificent edition *Flora Graeca Sibthorpiana* that circulated in 1825 [47], sixteen *Dianthus* taxa are cited (Supplementary Materials); an illustration (Figure 6)—based on an original watercolor by the painter Ferdinand Bauer (1760–1826) [48]—presents the flowering *Dianthus fruticosus* (plate 407), which corresponds to actual specimens collected from Serifos (Figure 6). A reference to *Dianthus fruticosus* (#974) is also cited on page 289 of the first volume of the edition *Florae Graecae Prodromus* [49], where it is written that “Sibthorp quotes the specimen from Serifos”. In addition, in a publication about the Botanic Garden in Oxford [50] that circulated in the mid nineteenth century, it was noted, “*Dianthus fruticosus* is a native of Greece, i.e., a shrub three feet in height with the flowers of a common pink of which there is a superb drawing in *Flora Graeca Sibthorpiana*”.

It is noteworthy that only 25 complete folio copies of the first edition of *Flora Graeca Sibthorpiana* were issued to subscribers from 1806 to 1840; a second edition of 40 copies was published between 1845 and 1856 [42,51,52]. The limited circulation of *Flora Graeca Sibthorpiana* minimized the possibility of access to this work, which provides a detailed insight into the complex process of recording rare plant specimens from Greek territories and writing a Flora in the early nineteenth century; at present, the output of the work of Sibthorp and his colleagues is well documented [42,52,53,54].

### 2.3. The Twentieth Century

A number of specimens brought into the U.K. by Sibthorp (in the late eighteenth century), which had been deposited in the Alexander Prior herbarium that was bequeathed to Kew in 1906, have been referenced as “ex Herb. Sibthorp” [55]; among these was “*Dianthus fruticosus* L. that Sibthorp quotes it from Serifos and Crete” [55]. These specimens were of considerable importance, not merely to Kew but as a supplement to Sibthorp’s plant collection at Oxford [56] have been presented officially to Oxford for incorporation in Sibthorp’s collection (Supplementary Materials). 

During the first half of the twentieth century, *Dianthus fruticosus* from Serifos was quoted in printed sources [57,58]. In addition, specimens of *Dianthus fruticosus* L. ssp. *fruticosus* collected from two islands of the Cyclades (Greece) were deposited in the Herbarium of the Goulandris Natural History Museum in Greece, i.e., specimens from Milos Island by H. Runemark and B. Bentzer (R. and Be. 29631) on 16 June 1967 and from Andros Island by E. Stamatiadou (6615) on 13 June 1969.

In 1980, the *Dianthus fruticosus* complex, consisting of obligate chasmophytes endemic to Southern Greece, was referred to a single species, *Dianthus fruticosus* L., subdivided into eight allopatric subspecies [14], i.e., *Dianthus fruticosus* ssp. *amorginus*, *D. fruticosus* ssp. *carpathus*, *D. fruticosus* ssp. *creticus*, *D. fruticosus* ssp. *fruticosus*, *D. fruticosus* ssp. *karavius*, *D. fruticosus* ssp. *occidentalis*, *D. fruticosus* ssp. *rhodius*, and *D. fruticosus* ssp. *sitiacus* (Supplementary Materials). In fact, Runemark noted that “within this complex it is possible to recognize a number of regional form series which may be of interest for the future study of the history of the Aegean Flora” [14].

**Figure 7:**
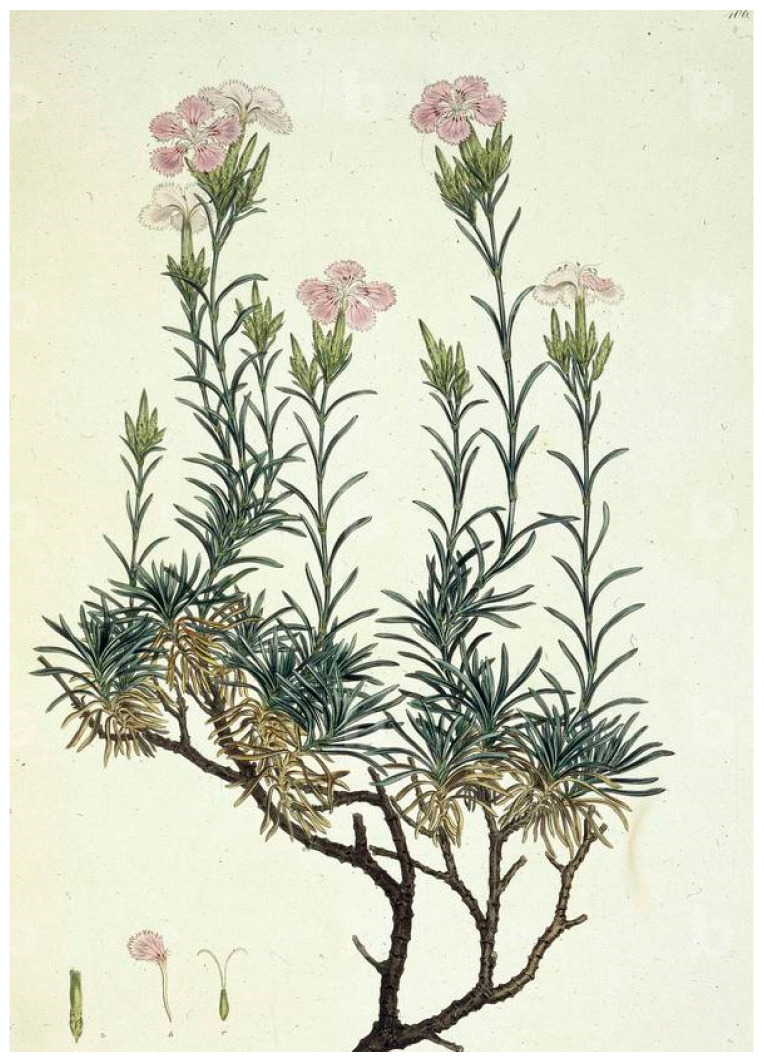
Illustration of *Dianthus arboreus* collected in a Greek territory in 1787 and published in *Flora Graeca Sibthorpiana*, based on the original watercolor by Ferdinand Bauer (reproduced with permission of the National Library of Greece).

*Dianthus arboreus* (Figure 7), which is a synonym of *Dianthus juniperinus* ssp. *bauhinorum* (Greuter) Turland [14,59], is also quoted as misapplied for *Dianthus fruticosus* L. [29]. In late twentieth century, it was argued that *Dianthus arboreus* was a name that many considered as a *nomen ambiguum*, i.e., name ambiguous [60]. Interestingly, Lack and Mabberley [52] cited this illustration (Figure 7) as “*Dianthus fruticosus* inscribed *Dianthus arboreus* Creta by James E. Smith” [52] (plate VII).

### 2.4. The Twenty-First Century

The distribution of *Dianthus fruticosus* L. ssp. *fruticosus* on the Cyclades Islands has been published [13] and is digitally available via Flora of Greece web [29]. Furthermore, ex situ laboratory experiments using plant material collected from Serifos [61,62] confirmed the occurrence of this indigenous, wild carnation on Serifos Island. Morphological traits of plant tissues of the endemic *Dianthus fruticosus* ssp. *fruticosus* have been reported in detail [14]. It has also been argued that scented and scentless flowers occur even within the same population on locus classicus for this subspecific taxon on Serifos Island [14], while the size of its petals varies between populations and even between flowers in the same inflorescence [14]. However, according to the best of our knowledge, the ecophysiological traits of the naturally occurring *Dianthus fruticosus* ssp. *fruticosus* on Serifos Island—which may contribute to underlying adaptive processes to the abiotic environmental conditions— have not hitherto been studied [24].

## 3. Discussion

Aspects of the vegetation history of the endemic *Dianthus fruticosus* L. ssp. *fruticosus* are discussed, with particular reference to archives and plant collections associated with the discovery of this subspecies on Serifos Island in the Cyclades (Greece).

The vascular flora of Serifos comprises approximately seven to eight hundred taxa [63,64,65,66]. The soil substrate of this island affects the distribution of plants to a large extent, and the occurrence of ores leads to unfavorable conditions for plant growth [67,68,69]. The long history of human impacts (agriculture, grazing, exploitation of iron deposits) has affected the flora of Serifos in different types of habitat. However, it seems likely that most of the plants recorded in 1928 [70] existed on the island in the beginning of the twenty-first century [63]. Moreover, the list of 286 taxa recorded on Serifos Island in the early twentieth century significantly increased in the early twenty-first century. The lower number of taxa cited in the flora of Serifos in 1928 is probably due not to observation gaps but to more intensive human activities during that period. Approximately 30 of the 286 taxa recorded on Serifos in 1928 [70] were not confirmed in the twenty-first century [63], perhaps overlooked because of either their seasonality or their unsuccessful establishment on the island. In addition, the data may reflect a lack of research.

Despite of the impacts and expansion of tourism [71,72], native species of Serifos have been preserved in small, undisturbed areas, cliffs, rocks, and steep ravines that serve as refugia. A large area of south Serifos is characterized as a Natura 2000 site (GR4220009, 37.142366° N, 24.463083° E) [73,74]; geomorphological features, flora and fauna, ancient ruins, and modern relics (abandoned mines) make Natura 2000 site GR4220009 of south Serifos of exceptional value. However, site GR4220009 is subjected to increasing pressure from tourism. Unfortunately, Natura site GR4220009 was exposed to an extended fire in June 2024 that destroyed the aboveground portion of all plant species in this area. In addition, the objective environmental and climatic changes that have occurred on the island over the years might induce a loss of species linked to more droughted habitats. At the same time, the reduction in cultivated plants in abandoned terraces due to tourism and water scarcity, which is a major environmental factor limiting plant development on Serifos Island, may favor the expansion of drought-tolerant native species—such as *Calicotome villosa*, *Capparis spinosa*, *Cichorium intybus*, *Dittrichia viscosa*, *Foeniculum vulgare*, *Lavandula stoechas*, *Pancratium maritimum*, *Pistacia lentiscus*, and *Sarcopoterium spinosum*—that have been there for a long period and adapted to abiotic and biotic conditions of inland and coastal sites.

The increased human-induced pressure on Serifos ecosystems—mainly related to touristic accommodation and associated activities—may negatively affect the species richness therein, as well as populations of *Dianthus fruticosus* L. ssp. *fruticosus*. Although some attempts at integrated plant biology—i.e., phytogeographical, biochemical, physiological, morphological, ecological, and molecular research; statistics; and conservation efforts—have been considered for large islands, relevant attempts for smaller islands in the Aegean archipelago are indeed rare [75,76,77].

When we seek to place botanical issues within their historical context, botanical explorations provide a seemingly convenient concept [78]. The process of bringing plant specimens from far-flung territories to a scientific and/or commercial center was an apt description of the eighteenth- and nineteenth-century botanical practices, which included collecting specimens and comprising accurate scientific illustrations in valuable publications such as in *Flora Graeca Sibthorpiana* [45,51]. This was a period of scientifically motivated botanical expeditions, of manufacturing specimens’ transfer boxes, of advances in printing techniques and increased wealth in Europe such that collections could be maintained and beautiful books could be produced [79,80,81]. Botanical explorations encompassed a wider range of aesthetic views and scientific knowledge [65,82,83], also shedding light on the desire for accurate representations of nature, mediated by an individual artist’s skill [33,34,36]. A botanical explorer (scientist, amateur, entrepreneur, eccentric) who invested in the science and practice of botany was also related to a wide range of motivations that influenced fielding botany, i.e., personal interests and choices, institutional agendas, risk and interactions with local agents, reliance on local knowledge, and strategies of self-promotion.

In the eighteenth century, major botanical expeditions were being undertaken by botanists from Europe to the eastern Mediterranean and the Levant. In that period, Greece was a botanically unexplored region and very dangerous to visit because of diseases, civil unrest, difficult transport, and bandit groups [27,51]. However, John Sibthorp and his colleagues explored Greek territories twice during the second half of the late eighteenth century (i.e., in 1786–1787 and 1794–1795) and collected wild plants grown under natural conditions, many of which were cited in the Herbal of Greek physician Pedanius Dioscorides, who lived in the Levant during the first century C.E. [84,85]. In fact, Sibthorp was aware of Dioscorides’s herbal and the medicinal properties of plants grown in the eastern Mediterranean before his botanical explorations to the Levant and the documentation of his plant collection [42,84,86]. One of Sibthorp’s goals was to find plants presented by Dioscorides and name them using the Linnaean binomial classification [42,87] that provided a lingua franca, facilitating scientific communication, long-distance comparisons, and determinations of plant specimens. It is noteworthy that Sibthorp travelled to Vienna to study the earliest illustrated manuscript related to Dioscorides’s work, written in Greek, dated to about 512 C.E., and known under various names, i.e., the Vienna Codex, Juliana Anicia Codex of Dioscorides, Codex Vindobonensis, and Codex Constantinopolitanus [40,88,89,90]. Elucidating Dioscorides’s work was quite important to early botanists.

The endemic carnation collected from Serifos Island in the early eighteenth century by Tournefort was labelled as Caryophyllus Graecus arboreus and Caryophyllus arboreus Seriphius in the form of pre-Linnean polynomials. A specimen of the endemic carnation collected from Serifos Island by Sibthorp in 1787 was cited as *Dianthus fruticosus* according to the Linnean binomial system in the fifth volume of *Flora Graeca Sibthorpiana* in 1825 [46]. Since 1980, the accepted scientific name for the indigenous carnation, which is still growing on Serifos Island, has been *Dianthus fruticosus* ssp. *fruticosus* [14,29].

The names of plants can be rich sources of specific and curious information; a plant name is also part of the cultural aspects of botany. Thus, the name *Caryophyllus* [91] used for the carnation is derived from the Greek word “karyophyllon” (καρυόφυλλον), which precisely means nut-leaved, i.e., phyllo (derived from the Greek word “φύλλο”, which means leaf) and caryo (derived from the Greek word “κάρυο”, which means nut) [91,92], and refers to of the spicy clove *Caryophyllus aromaticus* [91], which is native to Moluccas islands (Indonesia). To clarify the confusion, systematic botanists reclassified the spice clove as *Eugenia aromatica* and with the currently accepted scientific name *Syzygium aromaticum* [92]. *Caryophyllus* was the name of the clove-pink, the pink-tribe, i.e., a natural group of dicotyledons with flowers consisting of five petals [93]. The name *Dianthus* is derived from the two Greek words “dios” and “anthos” (διὸς and ἄνθος), meaning the flower of the ancient king of gods Zeus or Dios (Ζεύς and Διὸς, respectively in Greek), i.e., Dios’ flower [92,94]. It is noteworthy that the binomial nomenclature goes back to antiquity, because Linnaeus adopted the generic scientific name *Dianthus* from “Diosanthus” (διόσανθος), a name used by Theophrastus, who was known as the “Father of Botany”. In fact, Theophrastus was the first to name and describe the plant in the fourth century B.C.E. [95].

The genus *Dianthus* (carnation) is probably indigenous to the Mediterranean region, but its exact range is unknown because of extensive cultivation for the last 2000 years [7,96]. The common name “carnation” is probably derived from the old Italian word *carnagione*, meaning complexion (flesh color), or *corona*, i.e., a crownlike appendage, such as that on the inner side of a floral corolla of *Dianthus*’s flowers, used in ancient Greek and Roman ceremonial crowns (coronation) and wreaths. Carnations native to the southern and eastern Mediterranean region—also known with the vernacular names “garofano” in Italian, “clavel” in Spanish, “cravo” in Portuguese, “oeillet, girofle” in French, “karanfil” in Turkish, and “garyfallia” (γαρυφαλλιά) in Greek—have been introduced to other countries, where they have been cultivated, providing a large number of ornamentals known as florist’s gillyflowers [4,92,97]; the common English name “gillyflower” (also gillofre, gillyvor, and gilloflower) was applied to the carnation *Dianthus caryophyllus*, of which numerous cultivars exist [4,6,96]. To confuse matters even more, the term gillyflower had also been applied to wallflowers; the situation was clarified by naming pinks and carnations as “July flowers” because they were in full bloom during July [94]. The term “pink” referred to a rosy hue, named from a common floral color of the plant (i.e., pink). At the end of the eighteenth century, the pink joined the carnation as a florists’ flowering plant, and a noted center for both pinks and carnations was Oxford [94,96]. It is worth mentioning that Sibthorp returned to Oxford from his eastern Mediterranean explorations [98,99] carrying specimens for the Botanic Garden, but few details of these collections have survived; the plants, and any knowledge about their propagation, have been lost through many routes—including loss of willful destruction of records, poor note-keeping, and burning of garden records—and neglected [42,98].

Carnations pervade aspects of human life and have their own tales. People have created elaborate legends for the carnation that have been expressed in poetry, e.g., “The Gillyflower of Gold” and “Red Carnations” [100], and rituals, such as “if somebody sowed gillyflowers at noon on Good Friday, they would miraculously bear double blooms” [101]. Legends have also affected the symbolism of carnation, which is supposed to indicate bravery, love, pride, friendship, and energy [102].

**Figure 8:**
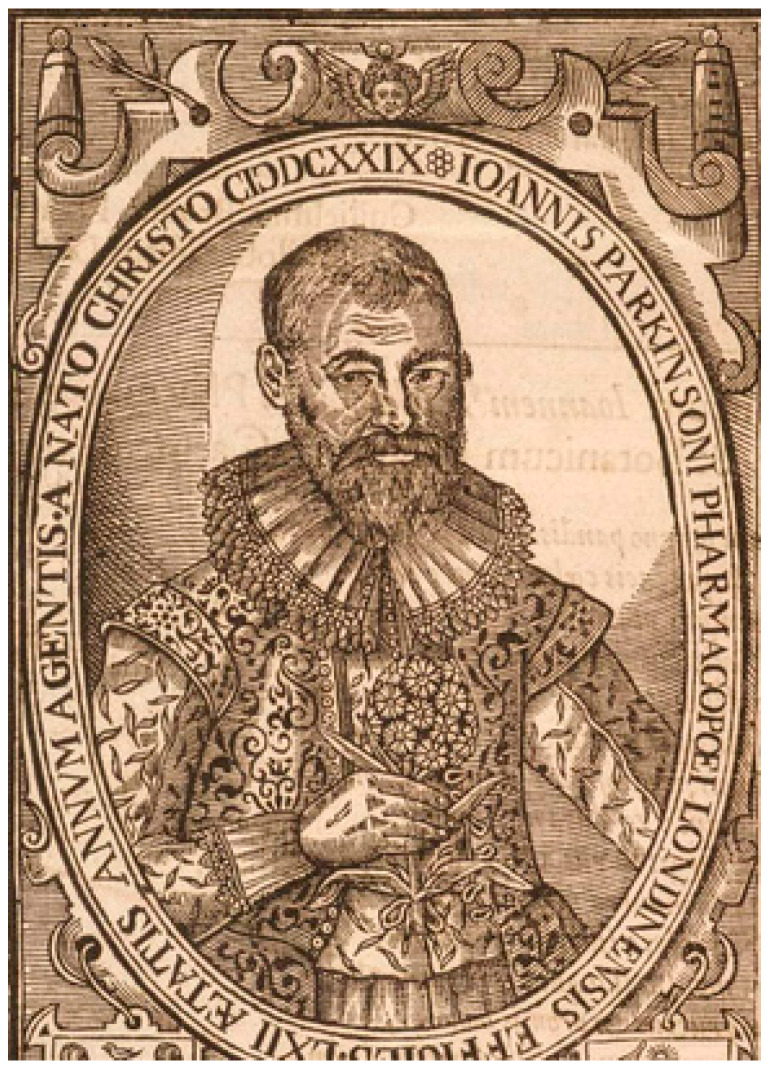
Portrait of John Parkinson; illustration from his *Paradisi in Sole Paradisus Terrestris* [106].

Archives and herbarium specimens of plants of symbolic and economic value to humans are related to ethnobotany as an assemblage of data, allowing research into both geographical and temporal changes in the occurrence of a particular species [103]. Herbarium specimens act as vouchers for the associated ethnobotanical information written on the herbarium sheets, enabling time and place to be specified [103]. Therefore, *Dianthus fruticosus* L. ssp. *fruticosus*’ specimens are indicative of samples’ collection in the context of botanical exploration in the eastern Mediterranean [42,65,82]. Ethnobotanical aspects also provide a motivation for gathering of data on the past medical uses of plants described in herbals, from classical to modern times. Information linked to the carnation was not found in Dioscorides’s primary source [104]. Interestingly, a portrait of the apothecary John Parkinson in 1629 (Figure 8) reveals that he is holding a flowering Sweet William (*Dianthus* species) that had no medicinal uses [105]; Parkinson (1567–1650), in his description of this plant [106], under the heading “The virtues”, states: “we have not known any of these used in Physicke”. According to the best of our knowledge, ethnomedicinal information connected to *Dianthus fruticosus* ssp. *fruticosus* is not hitherto available. However, medicinal properties—diuretic, sudorific, nervine stimulant, febrifuge and sedative—have been reported for flowers of other *Dianthus* species [102,107].

The natural distribution of the endemic *Dianthus fruticosus* ssp. *fruticosus* on the Cyclades islands of Greece—as well as that of *Dianthus fruticosus* ssp. *amorginus*, *D. fruticosus* ssp. *carpathus*, *D. fruticosus* ssp. *creticus*, *D. fruticosus* ssp. *karavius*, *D. fruticosus* ssp. *occidentalis*, *D. fruticosus* ssp. *rhodius*, and *D. fruticosus* ssp. *sitiacus*—has been published [13,14]. The long history of isolation, palaeogeographical patterns, and environmental morphological heterogeneity are factors known to affect the distribution patterns of endemic plants in this region [16,17,18,108,109,110,111]. The endemic *Dianthus fruticosus* ssp. *fruticosus* is part of a chain of plant life that spans the environment of Serifos and as such forms a basis of conservation measures. A more detailed study of its functional ecophysiological traits, coordinated with morphological features [14] that contribute to drought tolerance and the ecological niche [112], would help in constructing a clearer picture of the subject. The status of *Dianthus fruticosus* ssp. *fruticosus* on Serifos Island is both natural and cultural, involving complex relationships between this subspecific taxon and people over time.

## 4. Materials and Methods

### 4.1. Research Site

**Figure 9:**
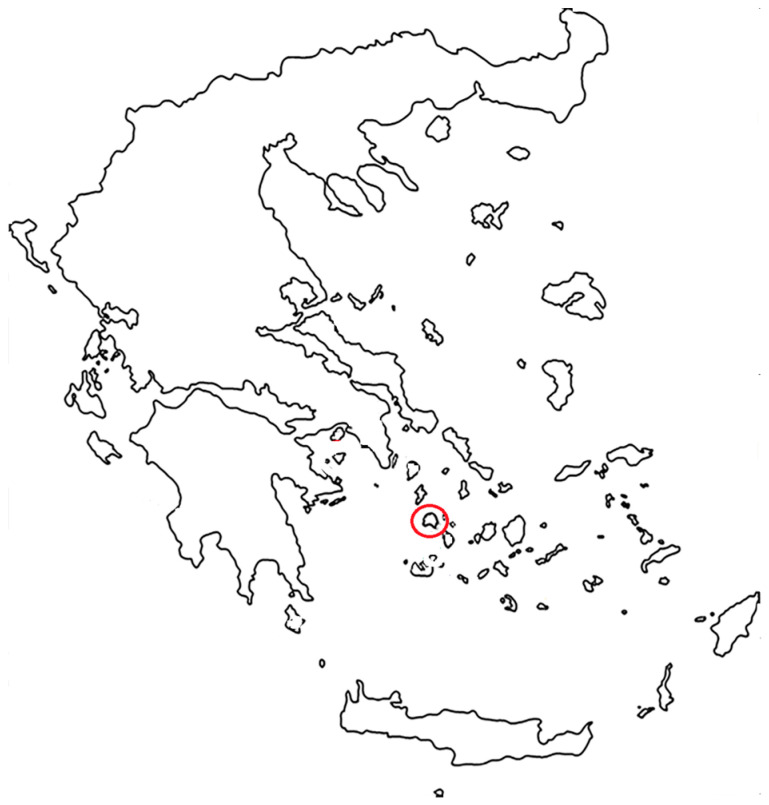
Abstract map of Greece, where the geographical location of Serifos Island is indicated by the red circle.

The Greek island of Serifos (37.1428° N 24.4974° E)—also mentioned in the literature, in ancient Greek texts and fragments, as Seriphos, Seriphus, Seriphi, and formerly Serpho [46,48,113,114,115]—is located in the western Cyclades in the Aegean archipelago (Figure 9). The area of Serifos is 75,207 km^2^, and the local population is approximately 1500 inhabitants. It is interesting to note that during the botanical explorations of the eighteenth century by Tournefort (in the year 1700) and Sibthorp (in the year 1787), the average local population of Serifos was approximately 450 inhabitants [116]. Plants collected on Serifos Island in those days correspond to “visual evidence” from a particular time, revealing regional, functional components of the biodiversity of this particular area, as well as the physical and aesthetic values of the natural environment.

In late nineteenth century, Serifos experienced modest economic development from the exploitation of iron ore deposits, porphyry, and green quartz crystals [67,68,117,118], contributing to the degradation of various habitats of the island. The mines ceased working in 1965, and currently, Serifos’s economy depends on tourism, infrastructure of country houses, and small-scale agriculture.

### 4.2. Climate

**Figure 10:**
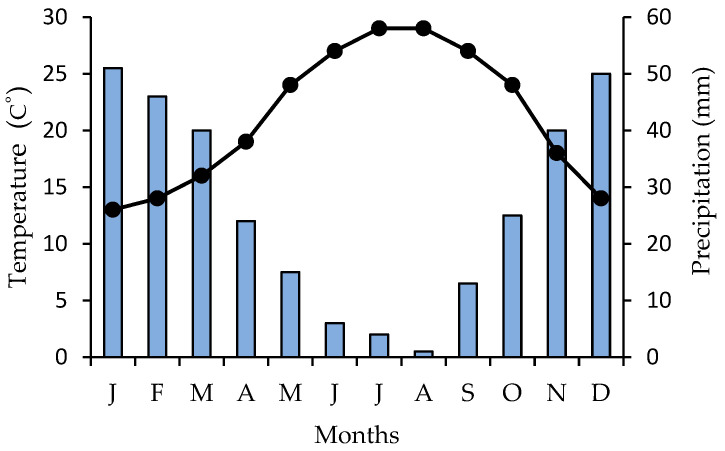
A multiannual ombrothermic diagram (precipitation scale = 2 × temperature scale) for the study site; the order of months is from January (J) to December (D). Mean monthly precipitation is indicated by blue bars, and mean monthly temperature, by closed circles and the black line.

The climate of the study site is Mediterranean with a marked seasonality; multiannual monthly climatic data (Figure 10) from 1999 to 2021, collected from a local weather station [119], indicate that the average air temperature is approximately 28 °C during the main flowering period of *Dianthus fruticosus* ssp. *fruticosus* from June to August, and the rainfall ranges from 4.0 mm to 0.5 mm, respectively.

### 4.3. Plant Material

The wild carnation *Dianthus fruticosus* ssp. *fruticosus* grows mainly in inland locations on Serifos (Cyclades, Greece) (Figure 9); it is a small shrub, with narrow, fleshy leaves and pinkish flowers, with a very long flowering period from June to October (Figure 2). We collected thirty specimens of *Dianthus fruticosus* ssp. *fruticosus* from shrubs of a rather large and growing population on cliffs near the Chora (the capital of Serifos, 200 m a.s.l., 37.1558° N, 24.5086° E), consistent with what the archives refer to [14,46,48]. A voucher specimen (accession number: Rhizopoulou s.n., 13.10.2024, ATHU) was deposited at the Herbarium of the National and Kapodistrian University of Athens in Greece. In fact, as has been published [14], the populations of *Dianthus fruticosus* ssp. *fruticosus* on Serifos Island are growing on siliceous rock and have purplish-pink flowers. *Dianthus fruticosus* ssp. *fruticosus* has been studied via archival research, written documents, and early plant collections dated from the pre-Linnean eighteenth century to the twenty-first century.

### 4.4. Archival Research 

The results were based on archival research, background, and information derived from the literature—i.e., via a thorough survey of written and online sources, plant catalogues, reports, books, and floras—related to *Dianthus fruticosus* ssp. *fruticosus*, such as: *Flora Graeca Sibthorpiana* [46], housed in the National Library of Greece (ΦΕ-2609); the Digital *Flora Graeca* [120,121]; *Florae Graecae Prodromus* [46], housed in the National and Kapodistrian University of Athens in Greece (M 433); the Herbaria at Oxford University in the U.K. [122]; and the Goulandris Natural History Museum in Greece. As the digital era is changing the use of libraries and archives, digital data have become subjects of investigation and contributed to a notable shift from closed to open in research and education, as an epistemological apparatus that allows specific production of knowledge [123]. The study of plant diversity in the context of climatic impact heightens the interest in archives [124,125,126].

## 5. Conclusions

*Dianthus fruticosus* L. ssp. *fruticosus* growing in exposed, vertical cliffs on Serifos Island attracted the interest of naturalists, visitors and scientists since the early eighteenth century. This review contributes to an appraisal investigation of natural history synthesizing knowledge from diverse findings for *Dianthus fruticosus* L. ssp. *fruticosus*; also, it is an example of how curiosity driven research can be turned into actionable knowledge. The status of the range-restricted, perennial *Dianthus fruticosus* L. ssp. *fruticosus* is associated with morphological and ecophysiological traits (underlying adaptive processes), and human-induced environmental activities. *Dianthus fruticosus* L. ssp. *fruticosus* is part of Serifos’ botanical heritage, linked to the environmental and historical conditions of this island.

## Data Availability

Data are contained within the article and supplementary materials.

## Supplementary Materials

The following supporting information can be downloaded at: https://www.mdpi.com/article/10.3390/plants13213002/s1.
