# Supplementary material for: Retrospective Approach to the Endemic Dianthus fruticosus L. ssp. fruticosus on Serifos Island (Cyclades, Greece)
Source: Plants (Basel). 2024 Oct 27;13(21):3002. doi: 10.3390/plants13213002 (PMC11548135; doi:10.3390/plants13213002)
Supplement: Supplementary file 1 [file plants-13-03002-s001.zip › plants-3225535-supplementary.pdf]

**File S1.** Species and subspecies of carnations found in Greek territories, as well as the endemic carnation of Serifos (in blue) as cited in selected, relevant publications referenced in the review.

---------------------------------------------------------------------------------

**Tourneforr[1] (1717)**
*Caryophyllus altilis major*
*Caryophyllus angustifolius*
*Caryophyllus arboreus Seriphius*
*Caryophyllus aromaticus*
*Caryophyllus eceruleus monspeliensis*
*Caryophyllus Graecus arboreus*
*Caryophyllus maximus variegatus*
*Caryophyllus sylvestris*
*Caryophyllus vulgaris*

---------------------------------------------------------------------------------

**Sibthorp and Smith[2] (1806)**
*Dianthus alpinus*
*Dianthus arboreus*
*Dianthus armeria*
*Dianthus biflorus*
*Dianthus caryophyllus*
*Dianthus carthusianorum*
*Dianthus cinnamomeus*
*Dianthus corymbosus*
*Dianthus crinitus*
*Dianthus diffusus*
*Dianthus fruticosus*
*Dianthus gracilis*
*Dianthus leucophaeus*
*Dianthus pinifolius*
*Dianthus pallens*
*Dianthus prolifer*
*Dianthus pubescens*
*Dianthus serratifolius*
*Dianthus strictus*
*Dianthus tripunctatus*

---------------------------------------------------------------------------------

**Sibthorp and Smith[3] (1825)**
*Dianthus arboreus*
*Dianthus biflorus*
*Dianthus carthusianum*
*Dianthus cinnamomeus*
*Dianthus corymbosus*
*Dianthus crinitis*
*Dianthus diffusus*

---

[1] Some *Caryophyllus* cited in: Tournefort, J.P. *Relation d'un voyage du Levant.* Lyons: Paris, France, 1717.
[2] *Dianthus'* taxa cited in: Sibthorp, J.; Smith, J.E. *Flora Graeca Prodromus: sive Plantarum Omnium Enumeratio, quas in Provinciis aut Insulis Graeciae*. London: Richard Taylor, 1806.
[3] *Dianthus'* taxa cited in: Sibthorp, J.; Smith, J.E. *Flora Graeca: sive plantarum rariorum historia, quas in provinciis aut insulis Graeciae*. London: Richard Taylor, 1825.

*Dianthus fruticosus*
*Dianthus gracilis*
*Dianthus leucophaeus*
*Dianthus pallens*
*Dianthus prolifer*
*Dianthus pubescens*
*Dianthus serratifolius*
*Dianthus strictus*
*Diantus tripunctatus*
--------------------------------------------------------------------------------

**Runemark[4] (1980)**
*Dianthus fruticosus*
*Dianthus fruticosus* ssp. *amorginus*
*Dianthus fruticosus* ssp. *carpathus*
*Dianthus fruticosus* ssp. *creticus*
*Dianthus fruticosus* ssp. *fruticosus*
*Dianthus fruticosus* ssp. *karavius*
*Dianthus fruticosus* ssp. *occidentalis*
*Dianthus fruticosus* ssp. *rhodius*
*Dianthus fruticosus* ssp. *sitiacus*
--------------------------------------------------------------------------------

**Georghiou & Delipetrou[5] (2010)**
*Dianthus androsaceus*
*Dianthus biflorus*
*Dianthus cinnamomeus*
*Dianthus cinnamomeus* ssp. *cinnamomeus*
*Dianthus cinnamomeus* ssp. *naxensis*
*Dianthus corymbosus*
*Dianthus desideratus*
*Dianthus diffusus*
*Dianthus fruticosus*
*Dianthus fruticosus* ssp. *amorginus*
*Dianthus fruticosus* ssp. *carpathus*
*Dianthus fruticosus* ssp. *creticus*
*Dianthus fruticosus* ssp. *fruticosus*
*Dianthus fruticosus* ssp. *karavius*
*Dianthus fruticosus* ssp. *occidentalis*
*Dianthus fruticosus* ssp. *rhodius*
*Dianthus fruticosus* ssp. *sitiacus*
*Dianthus gracilis* ssp. *xanthianus*
*Dianthus haematocalyx* ssp. *phitosianus*
*Dianthus haematocalyx* ssp. *pruinosus*
*Dianthus haematocalyx* ssp. *ventricosus*
*Dianthus juniperinus*
*Dianthus juniperinus* ssp. *aciphyllus*
*Dianthus juniperinus* ssp. *bauhinorum*

---

[4] *Dianthus fruticosus'* subspecies cited in: Runemark, H. Studies in the Aegean Flora XXIII. The *Dianthus fruticosus* complex (Caryophyllaceae). *Bot. Notiser*. **1980**, *133*: 475–490.
[5] Endemic *Dianthus'* species and subspecies cited in: Georghiou, K.; Delipetrou, P. Patterns and traits of the endemic plants of Greece. *Bot. J. Linn. Soc.* **2010**, *162*(2), 130–422.

*Dianthus juniperinus* ssp. *heldreichii*
*Dianthus juniperinus* ssp. *idaeus*
*Dianthus juniperinus* ssp. *juniperinus*
*Dianthus juniperinus* ssp. *kavusicus*
*Dianthus juniperinus* ssp. *pulviniformis*
*Dianthus mercurii*
*Dianthus myrtinervius* ssp. *caespitosus*
*Dianthus serratifolius*
*Dianthus serratifolius* ssp. *abbreviatus*
*Dianthus serratifolius* ssp. *serratifolius*
*Dianthus sphacioticus*
*Dianthus stamatiadae*
*Dianthus strymonis*
*Dianthus tymphresteus*
*Dianthus xylorrhizus*
--------------------------------------------------------------------------------

**Srtid[6] (2024)**
*Dianthus anatolicus*
*Dianthus androsaceus*
*Dianthus aridus*
*Dianthus armeria*
*Dianthus arpadianus*
*Dianthus biflorus*
*Dianthus cinnamomeus*
*Dianthus corymbosus*
*Dianthus crinitus*
*Dianthus cruentus*
*Dianthus deltoides*
*Dianthus deltoides* spp. *deltoides*
*Dianthus desideratus*
*Dianthus diffusus*
*Dianthus elegans*
*Dianthus formanekii*
*Dianthus fruticosus*
*Dianthus fruticosus* ssp. *fruticosus*
*Dianthus fruticosus* ssp. *amorginus*
*Dianthus fruticosus* ssp. *carpathus*
*Dianthus fruticosus* ssp. *creticus*
*Dianthus fruticosus* ssp. *karavius*
*Dianthus fruticosus* ssp. *occidentalis*
*Dianthus fruticosus* ssp. *rhodius*
*Dianthus fruticosus* ssp. *sitiacus*
*Dianthus giganteus*
*Dianthus glutinosus*
*Dianthus gracilis*
*Dianthus gracilis* spp. *gracilis*
*Dianthus gracilis* ssp. *drenowskianus*
*Dianthus gracilis* spp. *friwaldskyanus*

---

[6] *Dianthus'* species and subspecies cited in: Strid, A. *Atlas of the Hellenic Flora*; Broken Hill Publishers: Nicosia, Cyprus, 2024.

*Dianthus gracilis* spp. *xanthianus*
*Dianthus haematocalyx*
*Dianthus haematocalyx* spp. *haematocalyx*
*Dianthus haematocalyx* spp. *phitosianus*
*Dianthus haematocalyx* spp. *pindicola*
*Dianthus haematocalyx* spp. *pruinosus*
*Dianthus haematocalyx* spp. *ventricosus*
*Dianthus ingoldbyi*
*Dianthus integer* ssp. *minutiflorus*
*Dianthus juniperinus*
*Dianthus leucophoenicus*
*Dianthus mercurii*
*Dianthus monadelphus* ssp. *pallens*
*Dianthus muglensis*
*Dianthus myrtinervius* ssp. *myrtinervius*
*Dianthus myrtinervius* ssp. *caespitosus*
*Dianthus noeanus*
*Dianthus petraeus*
*Dianthus pinifolius*
*Dianthus pinifolius* ssp. *lilacinus*
*Dianthus pinifolius* ssp. *pinifolius*
*Dianthus pinifolius* ssp. *serbicus*
*Dianthus pinifolius* ssp. *tenuicaulis*
*Dianthus serratifolius*
*Dianthus simulans*
*Dianthus sphacioticus*
*Dianthus stamatiadae*
*Dianthus strictus*
*Dianthus strymonis*
*Dianthus superbus*
*Dianthus sylvestris*
*Dianthus tenuiflorus*
*Dianthus tripunctatus*
*Dianthus tymphresteus*
*Dianthus viscidus*
*Dianthus xylorrhizus*
*Dianthus zonatus*

---
